# Sleep Deprivation and the Epigenome

**DOI:** 10.3389/fncir.2018.00014

**Published:** 2018-02-27

**Authors:** Marie E. Gaine, Snehajyoti Chatterjee, Ted Abel

**Affiliations:** Department of Molecular Physiology and Biophysics, Iowa Neuroscience Institute, Carver College of Medicine, University of Iowa, Iowa City, IA, United States

**Keywords:** sleep deprivation, DNA methylation, histone modifications, microRNAs, long non-coding RNA

## Abstract

Sleep deprivation disrupts the lives of millions of people every day and has a profound impact on the molecular biology of the brain. These effects begin as changes within a neuron, at the DNA and RNA level, and result in alterations in neuronal plasticity and dysregulation of many cognitive functions including learning and memory. The epigenome plays a critical role in regulating gene expression in the context of memory storage. In this review article, we begin by describing the effects of epigenetic alterations on the regulation of gene expression, focusing on the most common epigenetic mechanisms: (i) DNA methylation; (ii) histone modifications; and (iii) non-coding RNAs. We then discuss evidence suggesting that sleep loss impacts the epigenome and that these epigenetic alterations might mediate the changes in cognition seen following disruption of sleep. The link between sleep and the epigenome is only beginning to be elucidated, but clear evidence exists that epigenetic alterations occur following sleep deprivation. In the future, these changes to the epigenome could be utilized as biomarkers of sleep loss or as therapeutic targets for sleep-related disorders.

## Introduction

Sleep is a fundamental requirement to maintain a healthy lifestyle and yet, millions of people across the world are sleep-deprived. In the United States alone, one-third of adults do not get sufficient sleep (Liu et al., 2016). Disruption of the classic sleep/wake cycle is commonly caused by staying awake due to work or lifestyle choices (Boivin and Boudreau, 2014). Sleep disruption can also be caused by the use of stimulants or health problems, including stress or sleep-related disorders (Drake et al., 2004, 2006).

The innate need for sleep is seen throughout evolution (Miyazaki et al., 2017) and its absence can cause a wide range of disturbances throughout the body. Sleep loss commonly disrupts metabolism, increases the risk of obesity, and has been associated with altered expression of metabolic genes and hormones (Skuladottir et al., 2016). Long-term comorbidities arising from sleep deprivation can include heart disease, stroke and high blood pressure (Javaheri et al., 2018). Additionally, indirect consequences associated with sleep loss, including increased accidents, are due to decreased alertness and changes in behavior (Boivin and Boudreau, 2014).

The brain is one of the organs most impacted by sleep or the lack thereof. Synaptic plasticity and the maintenance of synapse strength require sleep, and cognitive abilities, including learning and memory (Vecsey et al., 2015; Tudor et al., 2016), are impaired following sleep deprivation. Even a short period of sleep deprivation, 1 h after cognitive learning, can impair memory formation (Prince et al., 2014) and the hippocampal memory system appears to be especially sensitive to sleep loss (Havekes et al., 2016; Havekes and Abel, 2017). The alterations in the brain found following sleep deprivation can manifest as numerous phenotypes. These phenotypes can include altered mood states (Cote et al., 2013), the aggravation of certain psychiatric disorders (Wehr et al., 1987; Lewis et al., 2017) and neurodegenerative disorders like Alzheimer’s Disease due to increased accumulation of Amyloid-β (Kang et al., 2009).

The burden of sleep deprivation varies from person to person (Frey et al., 2004) suggesting that numerous biological and environmental factors contribute to how sleep deprivation affects an individual. One twin study showed that behavioral performance following sleep deprivation was highly heritable (Kuna et al., 2012), emphasizing a critical genetic component to sleep. Furthermore, dramatic gene expression changes have been found following sleep deprivation (Cirelli and Tononi, 1999; Cirelli et al., 2004; Vecsey et al., 2012) and certain gene expression patterns can be associated with levels of sensitivity to sleep deprivation (Arnardottir et al., 2014).

Gene expression is regulated by a variety of processes, including epigenetic mechanisms. Broadly, the term epigenetics refers to adaptations to the genome that do not alter the underlying genetic sequence. Specifically, epigenetic mechanisms control gene expression, and therefore the function of the cell, by modifying chromatin structure and accessibility of the genome (Brown et al., 2012; Espada and Esteller, 2013). There are three principal epigenetic mechanisms: (i) DNA methylation and hydroxymethylation; (ii) histone modifications; and (iii) non-coding RNAs (Espada and Esteller, 2013). The DNA modifications that occur as a consequence of these epigenetic mechanisms form the epigenome, which varies across cells and generations due to the fact that epigenetic modifications are dynamic and sensitive to their environment.

The impact of sleep deprivation on the epigenome has been studied in both animal and human model systems, but caveats in both systems are important to keep in mind when interpreting the results of these studies. Several sleep deprivation approaches have been developed for rodents (Havekes et al., 2012), allowing for control over the environment and the collection of specific brain tissue. However, an underlying problem of this model is that results identified in rodents may not parallel what is seen in humans. Studies of sleep deprivation in humans alleviate this issue but must be done in accessible samples, predominantly saliva or blood, and can be confounded by environmental factors. In addition, acute sleep deprivation studies may not entirely recreate the continual chronic wakefulness commonly seen in humans. Despite this, changes to the epigenome following chronic wakefulness are expected to resemble those changes observed after acute sleep deprivation, but would likely be more pronounced.

Chronically restricted sleep is an adverse environmental factor that affects a vast number of people, a consequence of living in an age of increasing access to technology, societal pressures and the expectation of long workdays. Consistent with this reality, our sleep environment can have a significant impact on the brain epigenome. Focusing on the epigenome can provide insight into the molecular mechanisms of sleep deprivation and generate biomarkers to identify prolonged-forced wakefulness and sensitivity to sleep loss. Further, identifying epigenetic changes that occur as a consequence of sleep deprivation could lead to the development of novel therapies to treat sleep-related disorders and the cognitive deficits associated with sleep loss. Biomarkers are biological changes that are easily quantified and associated with a phenotype; they have been employed to predict the presence, progression and therapeutic outcome of numerous disorders. Biomarkers have more therapeutic promise if their expression is consistent across multiple cell types as this allows identification through accessible samples like blood or saliva. Epigenetic changes that are consistent in both the brain and the blood of humans and mice have been observed for certain biomarkers. For example, increased DNA methylation in the brain-derived neurotrophic factor (*Bdnf*) gene has been associated with early-life environmental toxins in the brain and blood of mice, and in human cord blood (Kundakovic et al., 2015). Further, the methylation of a single base within the spindle and kinetochore associated complex subunit 2 (*SKA2*) gene has been proposed as a biomarker for suicidal behavior, with replication in brain and blood samples (Guintivano et al., 2014).

**Figure 1:**
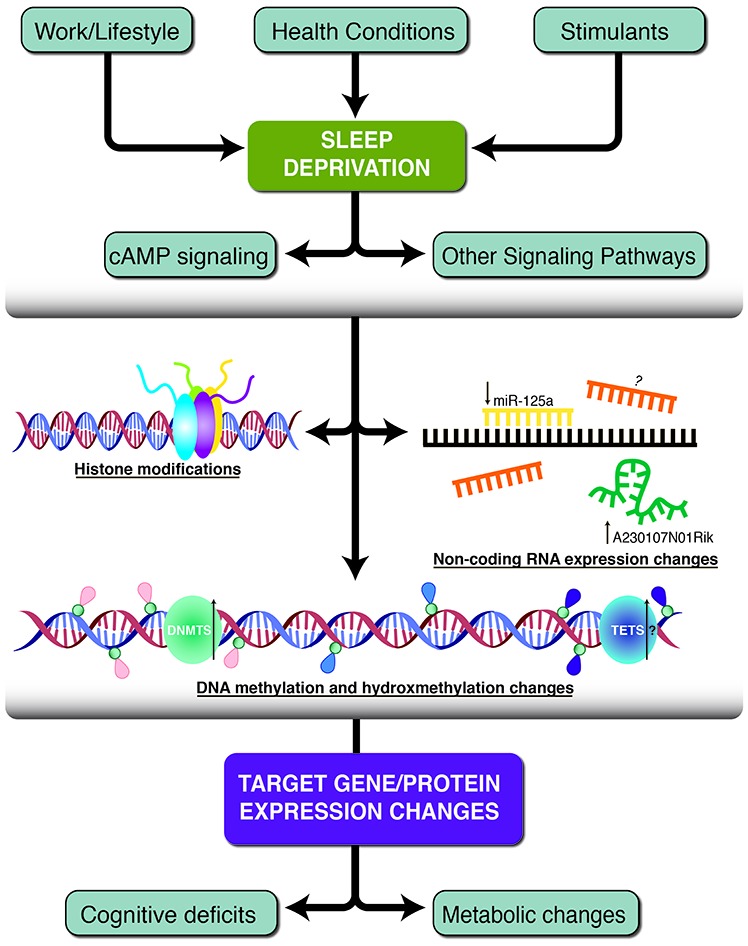
A schematic of the three primary epigenetic mechanisms found to be altered following sleep deprivation. Work and lifestyle, health conditions and stimulants can lead to sleep deprivation which causes subsequent neurobiological alterations. Histone modifications, non-coding RNA molecules and DNA methylation/hydroxymethylation work independently or together to change target gene and protein expression. Some of the resulting phenotypes include decreased cognitive deficits and metabolic changes. Pink tags represent methylated cytosines, blue tags represent unmethylated cytosines, and purple tags represent hydroxymethylated cytosines. The green and blue circles represent DNA methyltransferase (DNMT) and Ten-eleven translocation (TET) enzymes. Black arrows indicate the direction of expression, if known and additional question marks indicate speculative hypotheses without experimental evidence.

In this review article, we consider the link between sleep deprivation and the epigenetic landscape (Figure 1). We will focus on the epigenome; specifically, we will discuss the studies investigating DNA methylation, hydroxymethylation, histone modifications and non-coding RNAs following sleep deprivation, with a primary focus on acute sleep deprivation. It is important to note that, in addition to the information discussed here, there are numerous other avenues left to explore that link sleep deprivation with epigenetic modifications. This includes investigation into stimulants, medications, sleep-related disorders that may alter the epigenome in unique ways and the study of how chronic sleep loss differentially impacts the epigenome. Finally, we will discuss the current relationship between sleep and the epigenome as well as future directions for the continued study of the effects of sleep deprivation on epigenetic modifications.

## Sleep and the Methylome

The addition of a methyl group to a cytosine-guanine dinucleotide (CpG), termed DNA methylation, is considered the most common epigenetic modification. The DNA methylation patterns that occur throughout the genome are referred to as the methylome, and DNA methylation within the promoter region to the first exon of a gene generally correlates with decreased gene expression (Brenet et al., 2011). This decrease in gene expression occurs, in part, due to methyl-CpG binding protein 2 (MeCP2) which binds to the methylated DNA, alters the chromatin structure, and recruits transcriptional repressors to the methylation site (Nan et al., 1998; Martinowich et al., 2003). MeCP2 is abundant in the brain, indicating that functional DNA methylation is prevalent and essential for normal brain function and, when disrupted, may cause neurobiological changes.

DNA methylation levels are established and maintained throughout the genome by a family of enzymes called DNA methyltransferases (DNMTs), which include: DNMT1, DNMT2, DNMT3A, DNMT3B and DNMT3-like (DNMT3L). DNMT1 maintains the methylome during DNA replication (Espada and Esteller, 2013) and DNMT3A and DNMT3B are involved in the creation of *de novo* DNA methylation marks (Okano et al., 1999). DNMT3L has no enzymatic activity but is essential for DNA methylation during development (Espada and Esteller, 2013) and may be considered a partner of DNMT3A and DNMT3B. The function of DNMT2 is poorly understood, but it has the enzymatic ability to methylate DNA (Kaiser et al., 2016) and is also considered an RNA methyltransferase (Goll et al., 2006). The counterparts to the DNMTs are the Ten-eleven translocation (TET) enzymes: TET1, TET2 and TET3. These enzymes control DNA demethylation by adding a hydroxyl group to the methyl group, creating an intermediate, yet still functional base, 5-hydroxymethylcytosine (5hmC; Tahiliani et al., 2009). It is thought that 5hmC is particularly crucial in the brain due to its high abundance there compared to other organs (Li and Liu, 2011) and its enrichment in synapse-related genes (Khare et al., 2012). Furthermore, hydroxymethylation levels are enriched at exon-intron boundaries in the brain, which emphasizes a role for methylation in RNA splicing in these tissues (Khare et al., 2012).

There is substantial evidence to suggest that DNA methylation is critically affected by sleep. In mice, gene expression of *Dnmt3a1* and *Dnmt3a2* is increased following acute sleep deprivation, suggesting DNA methylation may be increased upon sleep loss (Massart et al., 2014). Further, twins with distinct diurnal preferences have different DNA methylation patterns (Wong et al., 2015) and short sleepers (<6.8 h) have altered DNA methylation patterns in 52 genes when compared to long sleepers (>7.8 h; Huang et al., 2017). In rats, deprivation of rapid eye movement (REM) sleep results in a significant change in the expression of genes related to DNA methylation (Narwade et al., 2017). There is also substantial evidence linking the methylation status of circadian clock genes and sleep loss (Qureshi and Mehler, 2014). One study investigated DNA methylation levels in males following one night of sleep deprivation (Cedernaes et al., 2015). They focused on circadian rhythm genes, including clock circadian regulator (*CLOCK*), cryptochrome circadian clock 1 (*CRY1*), brain and muscle ARNT-like 1 (*BMAL1*) and period circadian clock 1 (*PER1*). Two CpG sites in predicted *PER1* enhancer regions and one CpG site in the *CRY1* promoter region were found to be significantly hypermethylated in sleep-deprived adipose tissue samples. Notably, this did not correlate with a difference in gene expression. The lack of alterations in gene expression could be due to a delayed response, as suggested by the authors, intimating that gene expression does not immediately change following a difference in DNA methylation. It could also be due to the possibility that multiple CpG sites must be altered, or that other interactors, such as histone modifications and non-coding RNAs, are required in addition to DNA methylation in order to alter gene expression. The identification of DNA methylation changes in circadian rhythm genes (Cedernaes et al., 2015) extends on findings from a previous study that found hypermethylation of cryptochrome circadian clock 2 (*CRY2*) and hypomethylation of *CLOCK* in long-term night-shift workers (Zhu et al., 2011). These studies provide evidence that individuals subjected to acute sleep deprivation and those subjected to chronic sleep deprivation have comparable changes in their epigenetic landscape, at least with regard to circadian genes.

In addition to DNA methylation changes on circadian genes, altered DNA methylation patterns have been observed in genes involved in metabolism. Dysregulation of metabolic pathways is a common clinical outcome of sleep deprivation and metabolic enzymes regulate hippocampal memory (Mews et al., 2017), which is impacted by sleep deprivation. An enzyme critical in fatty acid desaturation, Stearoyl-CoA Desaturase 1 (*SCD1*), had increased DNA methylation near its transcription start site following sleep deprivation in male blood samples (Skuladottir et al., 2016). The previous studies show that DNA methylation changes can occur as a result of sleep deprivation, and may lead to the disruption of physiological processes such as metabolism and the regulation of circadian rhythms.

Hypothesis-driven approaches, like those mentioned previously, have the benefit of avoiding corrections for multiple testing, but will only add to the evidence regarding genes previously associated with sleep. Hypothesis-free approaches, like the genome-wide studies described below, are also required to identify novel genes or genomic regions associated with sleep loss. However, the vast amount of data generated can lead to errors. The first genome-wide study assessing the effects of acute sleep deprivation on DNA methylation was performed by Massart et al. (2014). Using custom arrays encompassing all promoter regions throughout the mouse genome, they compared the DNA methylation status of sleep-deprived and non-sleep-deprived male mice. A total of 227 probes showed altered DNA methylation following sleep deprivation and several of these correlated with changes in the expression of their target genes. One of the most significant changes was increased DNA methylation in an intron of the disks large homolog 4 (*Dlg4*) gene. The *Dlg4* gene encodes for the Postsynaptic Density Protein 95 (PSD-95), which forms scaffolds at excitatory synapses. Mice deficient in Dlg4 exhibit autism-like behaviors, such as increased repetitive and anxiety-like behaviors (Feyder et al., 2010). The increase in DNA methylation found in the intron of *Dlg4* corresponded with its increased expression, supporting the hypothesis that DNA methylation in the gene body positively correlates with gene expression. A genome-wide DNA methylation study using blood samples from healthy males, found 269 probes were significantly altered in sleep-deprived subjects (Nilsson et al., 2016). The main limitation of this study was the absence of supporting gene expression data. However, as a secondary analysis the authors correlated their findings to a previous study (Möller-Levet et al., 2013) to identify potential genes with altered expression and DNA methylation changes following sleep deprivation. One CpG site upstream of the inhibitor of growth 5 (*ING5*) gene had decreased DNA methylation with a similar decrease in expression of *ING5*. This gene encodes for a tumor suppressor but has also been associated with histone acetylation (Doyon et al., 2006), suggesting that various epigenetic mechanisms may work together to alter gene expression.

Hydroxymethylation levels have been studied at the genome-wide level following sleep deprivation (Massart et al., 2014). Because conventional bisulfite sequencing cannot distinguish between DNA methylation and hydroxymethylation, Massart et al. (2014) designed an array to specifically detect hydroxymethylation in all promoters, introns, and exons. Using a false discovery rate (FDR) of less than 0.10, they found sleep deprivation altered 5hmC patterns in 4697 genes. The regions identified were mainly exons and the 3′ untranslated region (UTR), which is dissimilar to typical DNA methylation patterns. Several synaptic adhesion genes had modified hydroxymethylation patterns following sleep deprivation, providing further evidence for the role of sleep in synaptic plasticity (Massart et al., 2014; O’Callaghan et al., 2017). Notably, hydroxymethylation may influence DNA methylation, as increased levels of hydroxymethylation were found at the 3’UTR of *Dnmt3a1* and *Dnmt3a2*, which correlated with increased expression of these genes following sleep deprivation.

Within specific genomic regions and throughout the genome, changes in DNA methylation and hydroxymethylation can be seen following sleep deprivation. These changes correlate with altered gene expression and help identify pathways dysregulated by sleep loss. Further work is required to define sleep-specific DNA methylation patterns, identify target genes affected by sleep deprivation, and determine the consequences of these changes.

## Involvement of Histone Acetylation in Sleep

Histone acetylation is the most extensively studied post-translational modification (PTM) in the nervous system (Peixoto and Abel, 2013). It is catalyzed by lysine acetyltransferases (HATs/KATs) that transfer an acetyl moiety from acetyl-CoA to lysine residues on a target protein. Lysine deacetylases (HDACs/KDACs) catalyze the reverse of this reaction. The dynamic interplay between HATs and HDACs results in the regulation of gene expression events that are critical for neuronal function. Histone acetylation weakens histone-DNA interactions resulting in a relaxed chromatin conformation, facilitating recruitment of transcriptional machinery. HATs have distinct preferences for certain substrates within the chromatin that marks the histones for specific events. Patterns of histone acetylation, in combination with other PTMs, are thought to serve as a code that regulates the signals for gene expression. Importantly, reduced expression or function of HATs may lead to neuronal dysfunction associated with decreased histone acetylation as is observed in several mouse models of neurodegenerative diseases and disrupted sleep-wake cycles in flies (Raggi and Ferri, 2010; Pirooznia et al., 2012).

Histone acetylation is critical for regulating gene expression and is related to various neurobiological mechanisms. Different forms of behavior and memory storage involve transcriptional and translational processes that occur during a specific time frame following a learning event (Hernandez and Abel, 2008; Peixoto et al., 2015). The transcriptional state of various genes is regulated by PTMs of histones and non-histone proteins and a recent report identified a link between metabolic enzymes, neuronal histone acetylation, and memory formation (Mews et al., 2017). Given the significant impact sleep deprivation has on gene expression, it is easy to surmise that sleep loss would alter histone modifications, thus affecting gene transcription.

**Figure 2:**
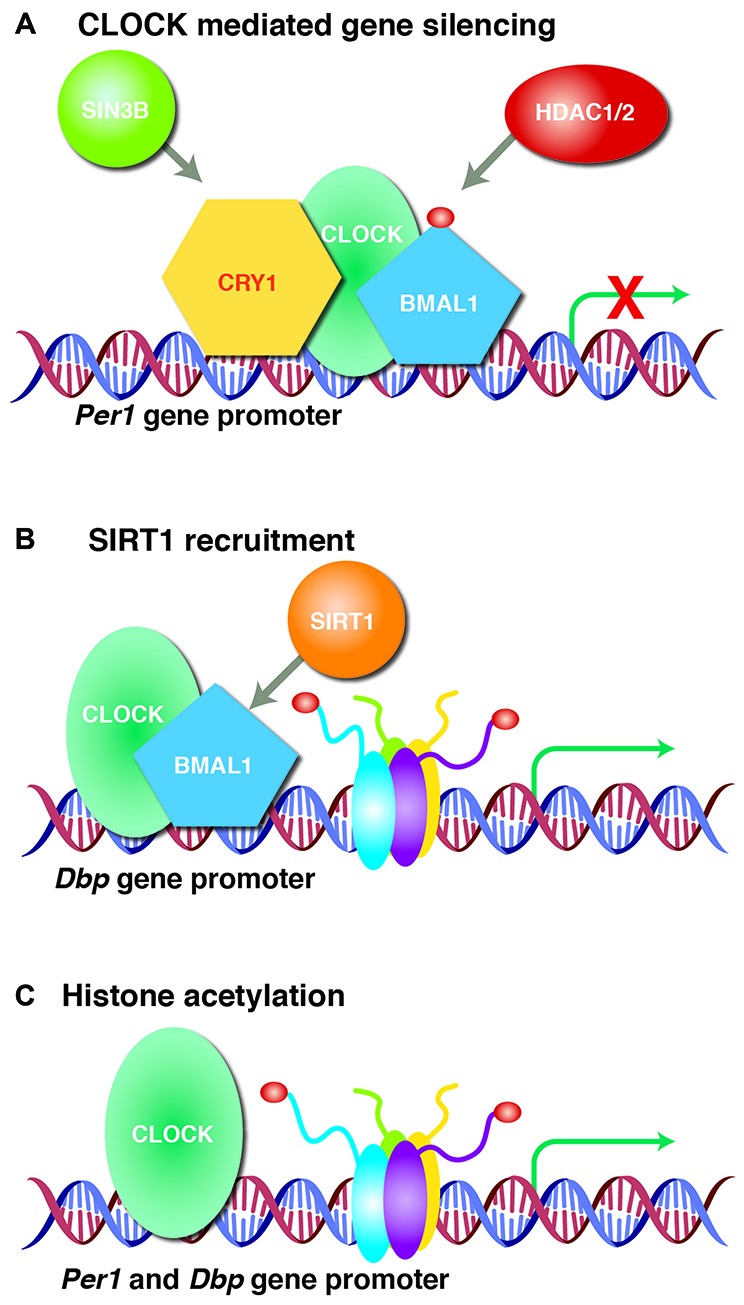
CLOCK regulates target gene expression. **(A)** Clock circadian regulator (CLOCK) acetylates brain and muscle ARNT-like 1 (BMAL1) and forms a heterodimer that recruits transcriptional silencer cryptochrome circadian clock 1 (CRY1) or SIN3 Transcription Regulator Family Member B (SIN3B) and histone deacetylase 1/2 (HDAC1/2) to silence period circadian clock 1 (*Per1*) gene expression. **(B)** CLOCK/BMAL1 heterodimer recruits sirtuin 1 (SIRT1) to the D-Box Binding PAR BZIP Transcription Factor (*Dbp*) promoter that rhythmically acetylates histones leading to periodic *Dbp* gene expression. **(C)** CLOCK itself rhythmically acetylates histones at *Per1* and *Dbp* gene promoters to regulate transcription. Acetylation is represented by dark red circles.

The circadian clock is thought to be closely linked to the epigenetic state of chromatin and cooperativity between HATs and HDACs tightly regulates the rhythmic transcription of clock-target genes. The CLOCK protein is a transcription factor and a histone acetyltransferase, with histones H3 and H4 being the primary substrates for acetylation (Doi et al., 2006; Hirayama et al., 2007), and is one of the most important regulators of genes related to circadian rhythm (Figure 2). In addition to its role as a histone acetyltransferase, the CLOCK protein can also directly acetylate proteins involved in circadian function, including BMAL1. The rhythmic acetylation of BMAL1 by CLOCK supports recruitment of the CRY1 protein to the CLOCK/BMAL1 heterodimer complex, promoting transcriptional silencing of CLOCK target genes, as has been shown in the liver (Hirayama et al., 2007). In addition, recruitment of CRY1 to the CLOCK/BMAL1 heterodimer complex silences transcription of the *Per1* gene by bringing the transcriptional co-repressor SIN3 transcription regulator family member B (SIN3B) and HDAC1/2 to its promoter (Naruse et al., 2004). The *Per1* promoter is also regulated by SIN3A and HDAC1, which are recruited by period circadian clock 2 (Per2) and polypyrimidine tract-binding protein-associated splicing factor (PSF; Duong et al., 2011). The CLOCK/BMAL1 heterodimer complex can also recruit sirtuin 1 (SIRT1) to facilitate rhythmic histone acetylation at the *Dbp* gene promoter leading to periodic expression of the *Dbp* transcript (Asher et al., 2008; Nakahata et al., 2008). Moreover, CLOCK rhythmically acetylates the *Per1* promoter and regulates circadian expression of *Per1* and D-box binding PAR BZIP transcription factor (*Dbp*) (Doi et al., 2006). CLOCK and BMAL1 also associate with transcription coactivators and acetyltransferases: cAMP responsive element binding protein (CREB)-binding protein (CBP), p300 and CBP-associated factor (PCAF; Takahata et al., 2000; Etchegaray et al., 2003; Curtis et al., 2004). Furthermore, mouse models in which circadian genes have been deleted display disrupted sleep homeostasis (von Schantz, 2008). Therefore, epigenetic mechanisms to modulate circadian genes are critical for the maintenance of circadian rhythm and sleep homeostasis.

The hippocampus is vulnerable to sleep deprivation (Prince and Abel, 2013; Havekes and Abel, 2017), which causes hippocampus-dependent spatial memory loss. This effect can be rescued upon treatment with the HDAC inhibitor, Trichostatin A (TSA; Duan et al., 2016). Recent evidence suggests that sleep deprivation reduces mRNA and protein levels of CBP HAT and increases levels of HDAC2, leading to a significant decrease in histone acetylation. *BDNF* gene transcription is a critical component of learning and memory, and is responsive to histone acetylation (Leal et al., 2015). Reduced occupancies of acetylated histones at the *BDNF* promoter IV have been observed in hippocampus samples from sleep-deprived rats, resulting in an impaired BDNF- Tropomyosin receptor kinase B (TrkB) signaling cascade (Duan et al., 2016). Another study also reported reduced *BDNF* transcription and translation along with decreased CREB expression after prolonged sleep deprivation (Guzman-Marin et al., 2006). These studies suggest that histone modifications, specifically in the hippocampus, are associated with sleep deprivation and cognitive deficits including memory impairment.

It is clear that the study of histone modifications associated with sleep is still in its infancy. The need for further exploration in this area will allow identification of precise epigenetic marks on the chromatin that are altered after sleep deprivation, enabling the identification of sleep-specific biomarkers or therapeutic targets for treating sleep-related disorders and the cognitive deficits associated with sleep loss.

## Sleep-Related Non-coding RNAs

It is an intriguing phenomenon that a substantial portion of the genome is made up of non-coding RNAs, although this is a controversial topic regarding how much of it is functional, rather than junk, RNA (Palazzo and Lee, 2015). These molecules are actively transcribed from DNA but typically do not code for proteins. They are several types of non-coding RNAs, but in the context of sleep, we will only discuss two. These non-coding RNAs can be grouped according to their size, the most common being long non-coding RNAs (lncRNAs) and microRNAs. Many have functions yet to be determined, but their abundance in normal and aberrant cells implicates a role for them in most biological pathways.

LncRNAs are larger than microRNAs (>200 base-pairs) and have many roles, including recruitment of epigenetic and regulatory components to target genomic loci, and regulation of splicing and translation (Shi et al., 2017). When located in the nucleus they modify chromatin structure and interact with chromatin-modifying enzymes to alter gene expression (Whitehead et al., 2009). An emerging area of research is in the ability of certain lncRNAs to encode small proteins, called micropeptides, challenging the theory that they are just non-coding molecules. Indeed, micropeptide-generating lncRNAs have been found in the brain (Mills et al., 2016). In addition, a large portion of tissue-specific lncRNAs are in the brain (Derrien et al., 2012) suggesting that they play an important role in neuronal function. For example, brain cytoplasmic RNA1 (*BC1*) acts as a translational repressor (Zhong et al., 2009). LncRNAs have also been linked to circadian machinery; deletion of the lncRNA *116HG* causes dysregulation of circadian genes *Clock*, *Cry1* and *Per2* (Powell et al., 2013). Using a mouse lncRNA array, differential expression of several lncRNAs was observed following sleep deprivation, with increased expression of *A230107N01Rik* being the most significant result (Davis et al., 2016). However, of the lncRNAs affected by sleep deprivation, there has currently been no associated function identified. It is important to note, especially in genome-wide studies, that expression of lncRNAs in most cell types is much lower than that of protein-coding transcripts (Derrien et al., 2012), making it difficult to reliably determine their expression in genome-wide experiments.

Mature microRNAs are single-stranded, small molecules (~22 nucleotides) predominantly found in the cytoplasm. MicroRNAs are first transcribed as longer primary transcripts, although some may be polycistronic (Sarnow et al., 2006). Primary transcripts are cleaved by Drosha to create precursor microRNAs. After translocation to the cytoplasm, precursor microRNAs are cleaved by Dicer to form the mature microRNA, which is loaded into the RNA-induced silencing complex (RISC). Mature microRNAs direct the RISC to bind to target seed sites. In most cases, incomplete complementarity to the target site results in repression of translation, while complete complementarity results in mRNA degradation through direct endonuclease cleavage (Krol et al., 2010). It was initially thought that microRNA binding sites resided in the 3’UTR of genes, but further research in the human brain has shown that microRNA binding sites can occur throughout the gene body (Boudreau et al., 2014). MicroRNAs are abundant in the brain, can be found within dendritic spines, and have been associated with many neurological processes, including synaptic plasticity (Lugli et al., 2008).

Several studies have shown that microRNAs are associated with circadian expression and sleep. This includes: CREB-regulated miR-132 and CLOCK/BMAL1-regulated miR-291-1, which have been associated with circadian timing (Cheng et al., 2007); the miR-192/194 cluster, which represses Per1–3 (Nagel et al., 2009); and miR-142-3p which may modulate BMAL1 protein expression (Shende et al., 2013). In addition, the circadian-related microRNAs, mir-132 (Davis et al., 2011) and miR-138 (Davis et al., 2012), have been associated with sleep loss in rats. Further, in depressed subjects with late insomnia, genetic variants have been found in the precursor miR-182, which may inhibit Adenylate Cyclase 6 (ADCY6), CLOCK and DSIP (also known as TSC22 Domain Family Member 3) expression (Saus et al., 2010). One genome-wide study performed to assess a link between microRNAs and sleep loss found let-7b and miR-125a were significantly altered in four brain regions following sleep deprivation (Davis et al., 2007). Numerous microRNAs were also found to be differentially expressed in specific brain regions, suggesting sleep may incur a tissue-specific regulation of microRNA expression. A subsequent microarray experiment in male mice sleep deprived for 6 h found enrichment of genes targeted by specific microRNAs and altered expression of 10 microRNAs (Mongrain et al., 2010). The altered expression of microRNAs after sleep loss is consistent with previous work showing that sleep deprivation inhibits mRNA processing and translation in the hippocampus (Vecsey et al., 2012). Sleep-related non-coding RNAs may be possible modulators of this impact on translation. Unfortunately, the abundance of predicted non-coding RNAs and their targets hinders the utility of hypothesis-driven experiments that could address this possibility. The implementation of deep RNA-sequencing analyses alongside validation through cloning will help identify non-coding RNAs associated with sleep.

## Conclusions and Future Directions

The prevalence of sleep deprivation throughout the world and the co-morbidity of sleep-related conditions with a wide range of diseases emphasize the importance of uncovering the molecular mechanisms related to sleep deprivation. This review aims to discuss the evidence that epigenetic mechanisms are altered following sleep deprivation, and to develop a framework that begins to delineate the effects of sleep loss on the epigenome. The evidence considered here suggests sleep deprivation is associated with several changes to the epigenome, particularly with regard to DNA methylation, histone modifications, and non-coding RNAs (Figure 1). It is important to note that the three epigenetic alterations discussed are not independent and may also regulate each other.

Details of how sleep deprivation causes these epigenetic changes are not fully understood. However, it is well known that sleep deprivation alters numerous signaling pathways, which could regulate epigenetic mechanisms. One example is the cyclic adenosine 3′,5′-monophosphate (cAMP) pathway, which is disrupted following sleep deprivation (Vecsey et al., 2009; Havekes et al., 2014, 2016). The cAMP response element-binding (CREB) protein mediates histone deacetylase inhibitors, suggesting that the cAMP pathway may alter histone modifications following sleep deprivation.

This review also highlights the infancy of studies on the effects of sleep deprivation on epigenetic modifications. One important unanswered question is if sleep recovery following deprivation can restore the epigenome to its initial state. To answer this question, future sleep studies should obtain samples after recovery from sleep deprivation. This will allow investigation into the fluidity of epigenetic marks following sleep deprivation, and determine if the changes are sustained or returned to baseline once sleep is restored. Another avenue to explore is the link between non-CpG methylation and sleep deprivation. The methylation of a cytosine preceding a base other than a guanine has been observed in both mouse and human brain samples (Xie et al., 2012; Lister et al., 2013) and becomes more abundant than methylated CpG sites in adult neurons. An additional interesting line of investigation would be RNA modifications, specifically methylation of N6-adenosine (m^6^A), which is emerging as a new type of epigenetic alteration. Decreased methylation of tRNAs causes tRNA fragments to activate cell stress response systems and repress translation (Blanco et al., 2014). Additionally, RNA methylation has been found in microRNAs (Berulava et al., 2015) and mRNAs as a label for degradation (Wang et al., 2014). Thus, we have only just begun to investigate the effects of sleep deprivation on the epigenome, leaving many exciting areas left to explore.

One other area to explore is the effects on the epigenome following the use of stimulants as a form of sleep deprivation. Two of the most common stimulants are caffeine and modafinil. Both can attenuate the negative effects of sleep deprivation (Wadhwa et al., 2015; Crawford et al., 2017) and promote prolonged wakefulness. Modafinil affects numerous neurotransmitter systems in the brain, including the dopaminergic system, by increasing extracellular levels of dopamine (Volkow et al., 2009) and can improve cognitive function (Minzenberg and Carter, 2008). In contrast, caffeine does not directly target the dopaminergic system, but blocks the neurotransmitter, adenosine, from binding to its receptor, resulting in improved cognitive function and increased alertness (Fredholm et al., 1999; Pasman et al., 2017). Therefore, understanding how stimulants may restore or alter the epigenome may shed further light on sleep-specific epigenetic modifications.

As the population continues to push the limits of prolonged wakefulness, the need to understand the molecular basis of sleep is becoming more prevalent. It is now well-known that sleep deprivation changes the transcriptome but how this occurs is not fully understood. The unique abundance of regulators of epigenetic mechanisms suggests that dysregulation of our epigenome could lead to altered synaptic plasticity and impaired learning and memory, as is observed in sleep-deprived individuals. Further epigenetic studies on the gene and genome-wide level are required to identify disrupted epigenomic regions that could predict therapeutic targets or potential biomarkers for the cognitive deficits caused by sleep deprivation.

## Author Contributions

TA proposed the outline and edited the manuscript. MEG and SC wrote the manuscript.

## Conflict of Interest Statement

The authors declare that the research was conducted in the absence of any commercial or financial relationships that could be construed as a potential conflict of interest.
